# Nonsyndromic Extraosseous Palate Neurofibroma of a 30-Year-Old Woman: A Case Report

**DOI:** 10.1155/crid/6778806

**Published:** 2025-04-02

**Authors:** Fahimeh Akhlaghi, Fatemeh Mashhadiabbas, Milad Baseri, Sanaz Gholami Toghchi, Maryam Mohammadalizadeh Chafjiri, Ardeshir Khorsand

**Affiliations:** ^1^Department of Oral and Maxillofacial Surgery, Dentistry Faculty, Shahid Beheshti University of Medical Sciences, Tehran, Iran; ^2^Department of Oral and Maxillofacial Pathology, Dentistry Faculty, Shahid Beheshti University of Medical Sciences, Tehran, Iran

**Keywords:** neurofibroma, palate, soft tissue, solitary

## Abstract

Neurofibroma (NF) is a benign peripheral nerve sheath tumor which consists of Schwann cells, perineurial-like cells, and fibroblasts. The NF can be central or peripheral, alone or multiple which is a manifestation of Type I neurofibromatosis. NFs are mostly found in the tongue, oral mucosa, and lips when appearing in the mouth cavity, but they are rare at palate in a solitary form. We report a case of a solitary NF originating in the hard palate in a 30-year-old woman. The patient had an asymptomatic, nonulcerated, sessile, pink, and smooth nodule measuring 15 × 25 × 5 mm with a firm consistency and defined border on the left side of the posterior part of the hard palate. She had a similar history about 12 years ago which was diagnosed as a benign myxoid tumor which was excised that time. The lesion was excised with a 3-mm safe margin. The periosteum was excised, but the palatal bone was intact and was not excised. IHC was positive for S-100 immunostaining. Eventually, the mass was diagnosed as a NF. The important point is that following the patient with NF is necessary, because it could be the primary sign of Type I neurofibromatosis. The follow-up of this patient is continuing, and until the accomplishment of this report, no relapse was evident.

## 1. Introduction

Neurofibroma (NF) is a benign peripheral nerve sheath tumor which consists of Schwann cells, perineurial-like cells, and fibroblasts. The NF can be central or peripheral, alone or multiple which is a manifestation of Type I neurofibromatosis. The head and neck site, especially the oral cavity, is rarely involved by the solitary type of NF [1]. Solitary NF (SN) is a rare variant of the neuroma without systemic and hereditary symptoms of NF1 [2]. NFs of the oral cavity often involve the trigeminal and upper cervical nerves [3]. NFs are mostly found in the tongue, oral mucosa, and lips when appearing in the mouth cavity [4]. NFs can occur in 10–70 years old, but most of the cases are in the 30s. The sex predilection of NFs remains a controversy [5]. The definitive cause of SN is still unknown. It is addressed that it usually is a hamartomatous malformation [6]. This article is about a rare case of SN on the hard palate.

## 2. Case Report

**Figure 1:**
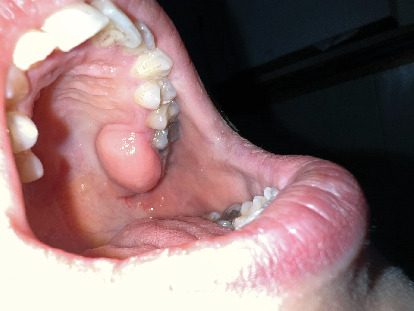
Presurgical clinical photo of the patient. The lesion is evident on the hard palate.

**Figure 2:**
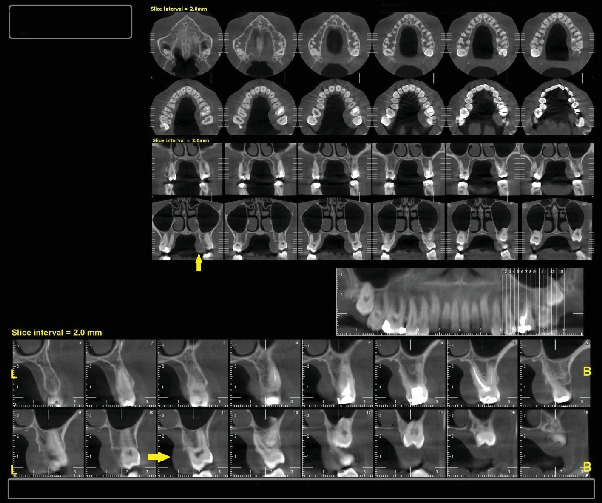
CBCT of the patient's maxilla in axial, coronal, and cross-sectional cuts. Yellow arrows show the soft tissue bulge in the palate with no invasion to sinus or nasal cavity; a mild corrosion of the lingual cortex of the maxillary alveolar bone is evident.

**Figure 3:**
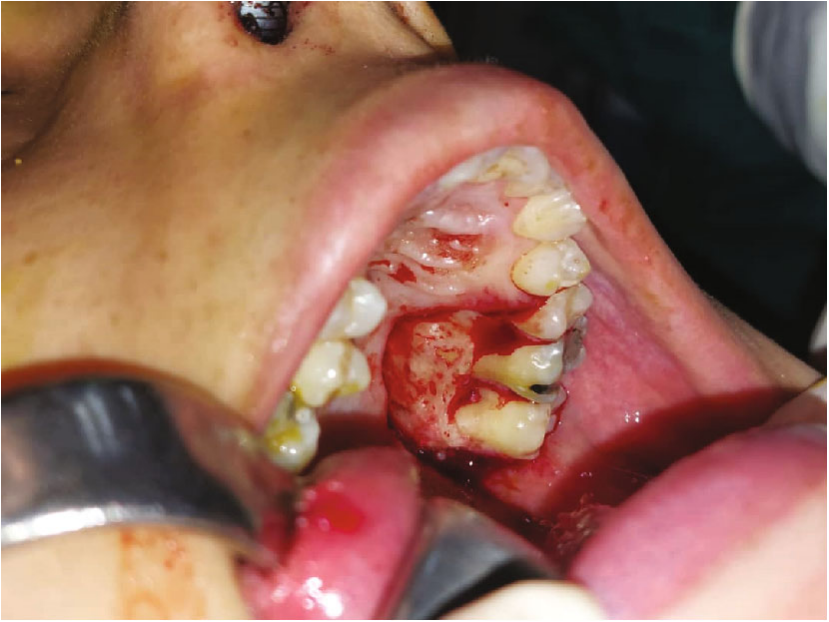
Postsurgical clinical photo of the patient. The lesion excised completely from the palate.

**Figure 4:**
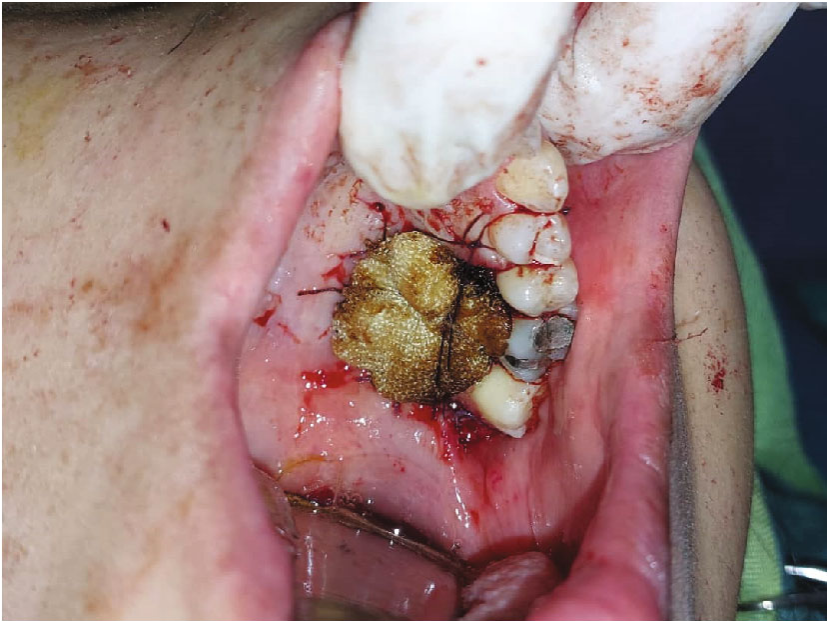
Surgical site with the oxidized cellulose in order to control bleeding.

**Figure 5:**
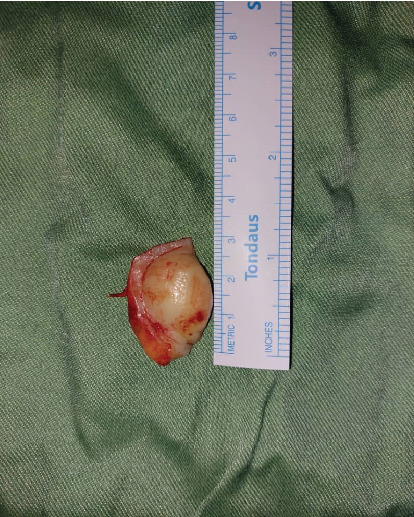
The specimen measuring 20 × 30 × 5 mm was referred to analysis in the medical pathology section.

A 30-year-old female was referred to Taleghani Hospital with a chief complaint of a lesion on the palate. She had a clear past medical history (no systemic disease such as diabetes or hypertension), habitual history (no smoking or alcohol), and familial history (no similar lesions in the family). In the clinical examination, the patient had an asymptomatic, nonulcerated, sessile, pink, and smooth nodule measuring 15 × 25 × 5 mm with a firm consistency and defined border on the left side of the posterior part of the hard palate, near the alveolar border (near the first and second molars) which did not cross the midline, that had been evolving for 3 years (Figure 1). No fluctuation was detected. The tongue was normal, and no lymph node enlargement was evident. There were no complaints such as dysphagia, odynophagia, dyspnea, uvula deviation, tonsilar involvement, nasal congestion, bleeding, hypoesthesia, or anesthesia in the palate. There was no mobility in the adjacent teeth, and they were vital. She had a similar lesion in the palate about 12 years ago which was diagnosed as a benign myxoid tumor which was excised that time. There were no signs of similar lesions on the other parts of the body. CBCT images showed a mass on the left side of the posterior site of the hard palate with a similar density to palatal soft tissue, extending from the upper left second premolar to the third molar. Also, the CBCT did not show any signs of invasion to the palatal bone by the lesion (Figure 2). The clinical diagnosis was soft tissue lesions; however, due to the previous surgery of the patient, the first line was benign myxoid tumor. Pleomorphic adenoma and benign mesenchymal neoplasia such as fibroma and fibromyxoma were other differential diagnoses. We did an excisional biopsy under general anesthesia with a 3-mm safe margin. The periosteum was excised, but the palatal bone was intact and was not excised. The greater palatine foramen was not involved in the lesion, and therefore, only the terminal branches of the greater palatine nerve were excised (Figure 3). In order to control bleeding, the oxidized cellulose was put in the site (Figure 4). The specimen measuring 30 × 20 × 5 mm, which was firm, with the same color as other parts of the palate, not hemorrhaging, and no part of necrosis, was referred to analysis in the medical pathology section in Taleghani Hospital (Figure 5). The histological diagnosis was soft tissue myxoma. However, for a precise investigation of the specimen, it was referred to the Oral and Maxillofacial Pathology Department in the dental faculty at Shahid Beheshti University of Medical Sciences.

**Figure 6:**
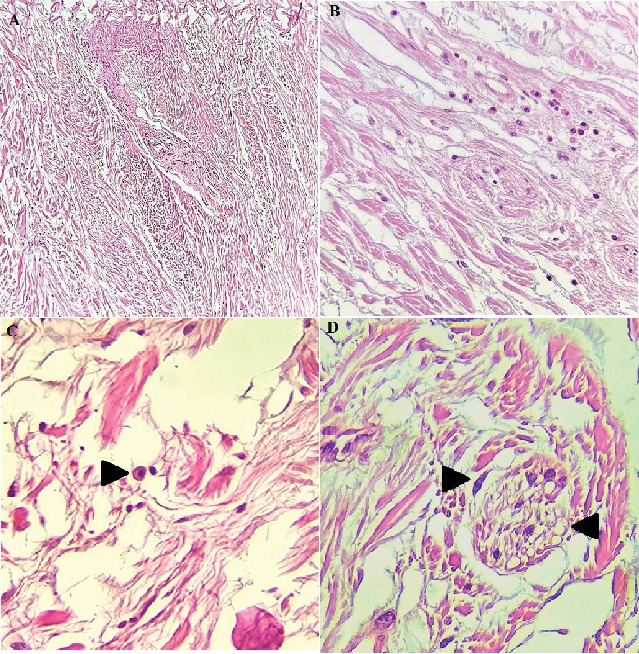
(A) Photomicrograph showing fibrous to myxoid connective tissue with abundant spindle cells with wavy nuclei, (B) giant fibroblasts and scattered chronic inflammatory cells infiltration, (C) the arrow shows the mast cell clearly, and (D) the arrow in the right shows wavy cells, and the left arrow shows the nerve bundle (H&E stain, ×100).

**Figure 7:**
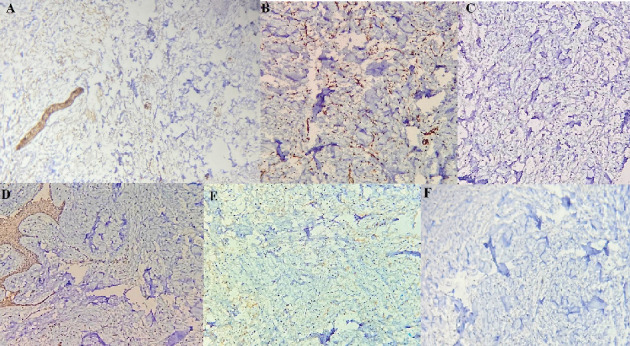
(A) Positive expression of S-100 (IHC, ×100). (B) Positive expression of CD34 (IHC, ×100). (C) Negative expression of SMA (IHC, ×100). (D) Negative expression of beta-catenin (IHC, ×100). (E) Negative expression of desmin (IHC, ×100). (F) Negative expression of STAT6 (IHC, ×100).

Histopathological sections showed fibrous to myxoid connective tissue, giant fibroblasts, lots of wavy nuclei-spindle cells, few mast cells, and infiltration of scattered chronic inflammatory cells which were representative of a mesenchymal tumor. Sections of muscle fibers, nerve bundles, minor salivary glands, adipose tissue, and reactive bone are evident. The lesion is covered by non- to parakeratinized stratified squamous epithelium (Figures 6A, 6B, and 6C). For precise diagnosis, immunohistochemical (IHC) staining for S-100 and CD34 was accomplished, which was positive (Figure 7). Also, IHC staining for SMA, desmin, beta-catenin, and STAT6 was negative. The final diagnosis was NF.

**Figure 8:**
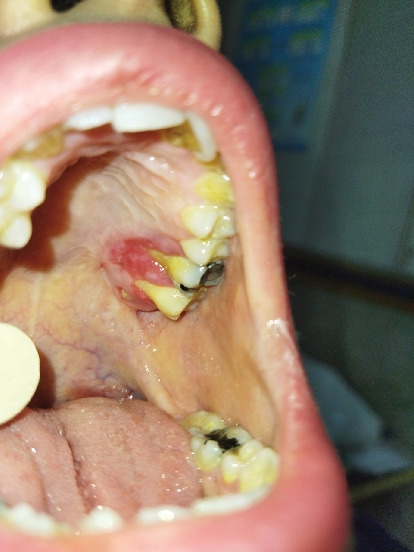
Clinical photo of the patient in follow-up after 3 months.

**Figure 9:**
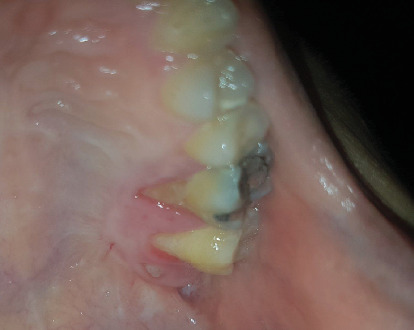
Clinical photo of the patient in follow-up after 6 months.

There were no signs of recurrence during the 3 months (Figure 8) and 6 months (Figure 9) of follow-up, and the area was showing the signs of epithelialization.

## 3. Discussion

According to the World Health Organization's 2020 classification [7], isolated, benign peripheral nerve tumors, not linked to NF1, can be classified as traumatic neuroma, solitary neuroma, and schwannoma [8]. NFs can manifest as a part of neurofibromatosis Type I (NF-I) or von Recklinghausen disease which present in approximately 1/3000 live births, commonly featuring café au lait spots on the skin and plexiform NFs in the peripheral or cranial nerves, which can transform into malignant peripheral nerve sheath tumors [9, 10]. Solitary fibrous tumors usually do not have a significant sex predominance [11].

The occurrence of SNFs in the oral cavity is rare, especially those unrelated to NF1, and is reported to have a frequency of 6.5% [12]. Solitary intraosseous NFs most commonly manifest in the posterior mandible, a tendency possibly attributed to the presence of long, thick bundles of the inferior alveolar nerve in that region [13]. SNFs are not common on the palate [14]. NFs contain high levels of signaling molecules such as chemokines, cytokines, and several growth factors which play a key role in the onset of pain in many neuropathic pain-like conditions [15], which in this case report, the patient had no pain. Our case closely resembled the hard palate SN described by Taketomi et al. in terms of macroscopic appearance [16]. In both instances, the lesion was round-ovoid, firm, and located at the left side of the posterior part of the hard palate. In the radiographic examination, we did not observe signs of invasion to the palatal bone which aligns with Taketomi et al.'s study [16].

Histopathologically, despite schwannomas exhibiting a well-defined capsule, NFs lack encapsulation. Also, spindle cell bundles with wavy nuclei in a collagenous stroma are seen in NF which are also S-100^+^ in most of the cases [3]. The S-100 protein is a useful marker for identifying nervous system tumors, and it is positive in both schwannoma and NF. In this case, S-100 was scattered positive [17] that aligns with Miettinen et al.'s study that reported IHC includes extensive but not diffuse S-100 positivity in NF spindle cell shape cells [18]. Generally, the discrimination between SNs and schwannomas is difficult [16]. Clinical history, radiographic, histopathological, and IHC evidences facilitate the diagnosis of NF [19]. CD34 is a sialylated transmembrane glycoprotein with an unclear function, expressed in myeloid progenitor cells, endothelial cells, and some fibroblast-like cells. Its tissue distribution is not fully understood. In tumor pathology, CD34 is particularly useful for differentiating between certain types of tumors. As shown by studies from Chaubal et al. [20] and Ohno et al. [21], NFs consistently contain a high number of CD34-positive spindle cells, whereas schwannomas do not. This distinct pattern of CD34 expression makes it a valuable marker for distinguishing NFs from schwannomas, which is why CD34 staining was performed in this case to aid in diagnosis and provide further insight into the tumor's cellular makeup.

The solitary fibrous tumor is characterized by an unencapsulated structure composed of spindle cells that are arranged in fascicles and a storiform arrangement. It typically exhibits positive staining for STAT6 and CD34; nevertheless, it is S-100 negative [22]. In this case, IHC staining for STAT6 was negative.

Leiomyoma, a benign tumor originating from smooth muscle, typically displays a swirled pattern of cells interspersed with vascular connective tissue. It usually stains positive for SMA and desmin while being negative for S-100 [23]. However, in this case, both SMA and desmin were negative. Histologically, fibromatosis is characterized by low-to-moderate cellularity, long fascicles of uniform cells, dense collagenous stroma, and a lack of malignant features. Usually, it exhibits nuclear *β*-catenin and SMA positivity [24]. Nevertheless, in this case, both *β*-catenin and SMA were negative.

Surgical excision is a recommended treatment plan for SN [25]. This case showed that there should be a significant attention to precise histopathologic evaluation because, on two occasions, the wrong pathologic report misled the surgeon, but finally the IHC technique report was accurate. The preferred treatment of SN is total excision of the lesion. However, due to the lack of encapsulation and the infiltrative nature of the lesion, recurrence is common. For preventing the recurrence, aggressive resection with safe margins, including the periosteum, is recommended [16]. Bleeding during surgery is the most prevalent complication [26] which was properly controlled by oxidized cellulose in our case.

## 4. Conclusion

In this case, we cannot claim that this lesion was a relapse of the previous lesion which was excised 12 years ago, but the most important point for clinicians is to follow the patient with NF even after excision of the lesions, because it could be the primary sign of Type I neurofibromatosis.

## Data Availability

Further data is available from the corresponding author on reasonable request.
